# Sustained HSP25 Expression Induces Clasmatodendrosis via ER Stress in the Rat Hippocampus

**DOI:** 10.3389/fncel.2017.00047

**Published:** 2017-02-22

**Authors:** Ji-Eun Kim, Hye-Won Hyun, Su-Ji Min, Tae-Cheon Kang

**Affiliations:** Department of Anatomy and Neurobiology, Institute of Epilepsy Research, College of Medicine, Hallym UniversityChuncheon, South Korea

**Keywords:** heat shock protein B1, status epilepticus, astrocyte, autophagy, apoptosis, ER stress

## Abstract

Heat shock protein (HSP) 25 (murine/rodent 25 kDa, human 27 kDa) is one of the major astroglial HSP families, which has a potent anti-apoptotic factor contributing to a higher resistance of astrocytes to the stressful condition. However, impaired removals of HSP25 decrease astroglial viability. In the present study, we investigated whether HSP25 is involved in astroglial apoptosis or clasmatodendrosis (autophagic astroglial death) in the rat hippocampus induced by status epilepticus (SE). Following SE, HSP25 expression was transiently increased in astrocytes within the dentate gyrus (DG), while it was sustained in CA1 astrocytes until 4 weeks after SE. HSP25 knockdown exacerbated SE-induced apoptotic astroglial degeneration, but mitigated clasmatodendrosis accompanied by abrogation of endoplasmic reticulum (ER) stress without changed seizure susceptibility or severity. These findings suggest that sustained HSP25 induction itself may result in clasmatodendrosis via prolonged ER stress. To the best of our knowledge, the present study demonstrates for the first time the double-edge properties of HSP25 in astroglial death induced by SE.

## Introduction

Astrocyte plays an important role in the maintenance of extracellular ion homeostasis and neuronal functionality in the brain. Since brain injury induces reactive astrogliosis representing astroglial hypertrophy, proliferation and migration, it is a general concept that astrocytes are invulnerable to various harmful stresses. However, a growing body of evidence indicates astroglial damage before or after neuronal damage and reactive astrogliosis (Sugawara et al., 2002; Borges et al., 2006; Kang et al., 2006; Kim D. S. et al., 2008; Kim J. E. et al., 2008; Kim et al., 2014a).

Status epilepticus (SE) is defined as continuous unremitting seizure activity longer than 5 min. SE is the most extreme form of seizure with significant mortality, and causes severe brain damage that may involve epileptogenesis (Kang et al., 2006; Kim J. E. et al., 2008; Trinka et al., 2012). Interestingly, SE evokes three different patterns of astroglial death in the rat brain independent of hemodynamics (Kang et al., 2006; Kim J. E. et al., 2008; Kim et al., 2010a,b, 2014a; Ryu et al., 2011a,b). One is focal necrotic astroglial degeneration detected in the piriform cortex accompanied by severe vasogenic edema 1–2 days after SE (Kim et al., 2013, 2014b), similar to other brain regions (Ingvar et al., 1994; Schmidt-Kastner and Ingvar, 1994; Gualtieri et al., 2012). Another is terminal deoxynucleotidyl transferase dUTP nick end labeling (TUNEL)-positive astroglial apoptosis, which is observed in the molecular layer (not the hilus) of the dentate gyrus (DG; Kang et al., 2006; Kim D. S. et al., 2008; Kim et al., 2010b, 2015). The other is clasmatodendrosis that is observed in the CA1 region (Kang et al., 2006; Kim D. S. et al., 2008; Kim et al., 2010b; Ko et al., 2016). Clasmatodendrosis is characterized by extensive swollen vacuolized cell bodies and disintegrated/beaded processes, which is first reported by Alzheimer (1910), and termed “clasmatodendrosis” by Cajal (Penfield, 1928). In addition, clasmatodendritic astrocyte has round-shaped edematous cell body, short blunt processes, GFAP tangles in the cytoplasm and nuclear dissolution. This irreversible astroglial change indicates impairment of astroglial functions. Vacuolization and eosinophilic cytoplasm in clasmatodendritic astrocytes make clasmatodendrosis be considered as coagulative necrotic astroglial death (Sugawara et al., 2002; Kim D. S. et al., 2008; Kim et al., 2009). However, we have reported that vacuolization in clasmatodendritic astrocytes is relevant to up-regulation of lysosome-associated membrane protein 1 (LAMP1), which is required for the essential activation of autophagy. Thus, clasmatodendrosis is an autophagic astroglial death (Ryu et al., 2011a,b). However, less defined are the molecular information available to clasmatodendrosis, although the distinct mechanisms or the heterogeneous properties of astrocytes seem to be relevant to clasmatodendrosis in the CA1 region.

Heat shock protein (HSP) 25 (murine/rodent 25 kDa, human 27 kDa) constitutes one of the major astroglial HSP families and plays a protective role against cell death (Cuesta et al., 2000; Bidmon et al., 2008; Lively and Brown, 2008). Therefore, HSP25 induction provides insight into the astroglial invulnerability in response to harmful insults. Furthermore, HSP25 induction is an indicative of the early astroglial responses including energy-consuming protein synthesis (Cuesta et al., 2000; Kirschstein et al., 2012). Thus, HSP25 is one of the highly sensitive and reliable molecules of full development of SE (Kirschstein et al., 2012). Recently, we have reported that endoplasmic reticulum (ER) stress induced by accumulation of unfolded or misfolded protein aggregates (Bernales et al., 2006) is relevant to astroglial apoptosis and clasmatodendrosis following SE (Ko et al., 2015). Because HSP25 binds to misfolded proteins and inhibits protein aggregation (Haslbeck, 2002; Richter-Landsberg, 2007), these findings had encouraged us to speculate that HSP25 induction would protect astrocytes from SE-induced ER stress. Unexpectedly, in the present study we found that SE-induced prolonged HSP25 expression resulted in ER stress and subsequently provoked clasmatodendrosis in the CA1 region *in vivo*. Thus, we demonstrate for the first time that sustained HSP25 induction itself may play a pro-autophagic factor in astrocytes via prolonged ER stress.

## Materials and Methods

### Experimental Animals and Chemicals

This study utilized the progeny of male Sprague-Dawley (SD) rats (7 weeks old) obtained from Experimental Animal Center, Hallym University (Chuncheon, South Korea). The animals were provided with a commercial diet and water *ad libitum* under controlled temperature, humidity and lighting conditions (22 ± 2°C, 55 ± 5% and a 12:12 light/dark cycle with lights). Experimental procedures were approved by the Institutional Animal Care and Use Committee of the Hallym university (Chuncheon, South Korea). The number of animals used and their suffering was minimized in all cases. All reagents were obtained from Sigma-Aldrich (USA), unless otherwise noted.

### Surgery and HSP25 Knockdown

Animals were anesthetized with 1–2% Isoflurane in O_2_ and placed in a stereotaxic frame. Animals were then implanted with a brain infusion kit 1 (Alzet, Cupertino, CA, USA) into the right lateral ventricle (1 mm posterior; 1.5 mm lateral; −3.5 mm depth). The infusion kit was sealed with dental cement and connected to an osmotic pump (1007D, Alzet, Cupertino, CA, USA) containing control siRNA or HSP25 siRNA. For knockdown of HSP25, we applied a set of four on-target HSP25 rat siRNAs and a non-targeting control was used in the preliminary study. Of four on-target siRNAs, a siRNA sequence corresponding to coding region (sense 5-GGAACAGUCUGGAGCCAAGUU-3; antisense 5-CUUGGCUCCAGACUGUUCCUU-3) was selected as the best probe (reduction efficiency: 43.2%, Genolution Pharmaceuticals, Inc., South Korea), and used for the final experiments. A siRNA sequence coding region 5-GCAACUAACUUCGUUAGAAUCGUUAUU-3 was used as a non-targeting control siRNA. The pump was placed in a subcutaneous pocket in the dorsal region. Animals received 0.5 μl/h of saline or (100 μM in saline) for 1 week (Kim et al., 2011, 2014b). Some animals were also implanted with monopolar stainless steel electrode (Plastics One, Roanoke, VA, USA) into the left dorsal hippocampus (−3.8 mm posterior; 2.0 mm lateral; −2.6 mm depth). Connecting wire and electrode socket were then inserted in an electrode pedestal (Plastics One, Roanoke, VA, USA), secured to the exposed skull with dental acrylic. Three days after surgery, rats were induced SE by LiCl-pilocarpine.

### SE Induction and EEG Analysis

Animals were given LiCl (3 mEq/kg, i.p) 24 h before the pilocarpine treatment. To reduce the peripheral effects of pilocarpine, atropine methylbromide (5 mg/kg, i.p.) was also received 20 min before the pilocarpine treatment. To induce SE, animals were treated with pilocarpine (30 mg/kg, i.p.). Control animals received saline in place of pilocarpine. For evaluation of the effect of siRNA knockdown on seizure susceptibility in response to LiCl-pilocarpine, animals were recorded EEG signals with a DAM 80 differential amplifier (0.1–3000 Hz bandpass; World Precision Instruments, Sarasota, FL, USA). EEG activity was measured during the 2 h recording session from each animal. The data were digitized (400 Hz) and analyzed using LabChart Pro v7 (AD Instruments, Bella Vista, NSW, Australia). Time of seizure onset was defined as the time point showing paroxysmal depolarizing shift, which lasted more than 3 s and consisted of a rhythmic discharge between 4 and 10 Hz with amplitude of at least two times higher than the baseline EEG (Kim and Kang, 2011). EEG activity was measured during the 2 h recording session from each animal. Spectrograms were also automatically calculated using a Hanning sliding window with 50% overlap. Two hours after SE onset, diazepam (Valium; Roche, France; 10 mg/kg, i.p.) was administered and repeated, as needed.

### Tissue Processing

Animals were perfused transcardially with phosphate-buffered saline (PBS, pH 7.4) followed by 4% paraformaldehyde in 0.1 M phosphate buffer (PB, pH 7.4) under urethane anesthesia (1.5 g/kg i.p.). The brains were removed, postfixed in the same fixative for 4 h and rinsed in PB containing 30% sucrose at 4°C for 2 days. Thereafter the tissues were frozen and sectioned with a cryostat at 30 μm and consecutive sections were collected in six-well plates containing PBS. For western blot (WB), animals were decapitated under urethane anesthesia. The hippocampus was rapidly removed and homogenized in lysis buffer. The protein concentration in the supernatant was determined using a Micro BCA Protein Assay Kit (Pierce Chemical, Rockford, IL USA).

### Double Immunofluorescence Study

Table 1 is a list of the primary antibodies used in the present study. Sections were incubated in a mixture of antisera in PBS containing 0.3% Triton X-100 overnight at room temperature. After washing, sections were incubated in a mixture of FITC- and Cy3-conjugated IgG (or streptavidin, Jackson Immunoresearch Laboratories Inc., West Grove, PA, USA; diluted 1:250) for 2 h at room temperature. Images were captured using an AxioImage M2 microscope. Fluorescent intensity was measured using computer-based image analysis program (AxioVision Rel. 4.8 software, Germany). Fluorescent intensity was then standardized by setting the threshold level (mean background intensity obtained from five image input). Manipulation of the images was restricted to threshold and brightness adjustments to the whole image.

**Table 1:** **Primary antibodies used in the present study**.

Antigen	Host	Manufacturer (Catalog number)	Dilution used
β-actin	Mouse	Sigma (A5316)	1:5000 (WB)
CHOP	Rabbit	Abcam (ab179823)	1:100 (IF)
CNX	Rabbit	Abcam (ab22595)	1:250 (IF)
GFAP	Mouse	Millipore (MAB3402)	1:5000 (IF)
GRP78	Rabbit	Thermo (PA1-37805)	1:100 (IF)
HSP25	Rabbit	Enzo (ADI-SPA-801)	1:500 (WB)
			1:500 (IF)
LAMP1	Rabbit	Abcam (ab24170)	1:100 (IF)
LC3-II	Rabbit	Abgent (AP1802a)	1:100 (IF)
PDI	Mouse	Abcam (ab2792)	1:250 (IF)
peIF2A	Rabbit	Sigma (SAB4504388)	1:200 (IF)
pPERK	Rabbit	Biorbyt (orb6693)	1:250 (IF)

*IF, Immunofluorescence; WB, Western blot*.

### TUNEL Staining

TUNEL staining was performed with the TUNEL apoptosis detection kit (Merck Millipore, Billerica, MA, USA) according to the manufacturer’s instructions. Following TUNEL reaction, GFAP immunofluorescence (IF) staining was performed. For nuclei counterstaining, we used Vectashield mounting medium with DAPI (Vector, Torrance, CA, USA).

### Cell Counts

For quantification of immunohistochemical data, cells in 2–4 regions (1 × 10^5^ μm^2^) from each section were counted on 20× images. Results are presented as means ± SD of 15–24 regions from seven animals. All immunoreactive cells were counted regardless of the intensity of labeling. Cell counts were performed by two different investigators who were blind to the classification of tissues.

### Western Blot

Aliquots containing 20 μg total protein were loaded into a polyacrylamide gel. After electrophoresis, gels were transferred to nitrocellulose transfer membranes (Schleicher and Schuell BioScience Inc., Keene, NH, USA). To reduce background staining, membranes were incubated with 5% nonfat dry milk in Tris-buffered saline containing 0.1% Tween 20 for 45 min, followed by incubation with the primary antibody (Table 1), and subsequently with an HRP-conjugated secondary antibody. Immunobands were detected with an ECL Western Blotting Detection Kit (Amersham, Piscataway, NJ, USA). Intensity measurements were represented as the mean gray-scale value on a 256 gray-level scale (Kim and Kang, 2011).

### Quantification of Data and Statistical Analysis

All data obtained from the quantitative measurements were analyzed using Mann-Whitney or Kruskal-Wallis test to determine statistical significance. Bonferroni’s test was used for *post hoc* comparisons. A *p*-value below 0.05 was considered statistically significant (Kim and Kang, 2011; Kim et al., 2014a).

## Results

### Regional Specific HSP25 Induction in the Hippocampus Following SE

**Figure 1:**
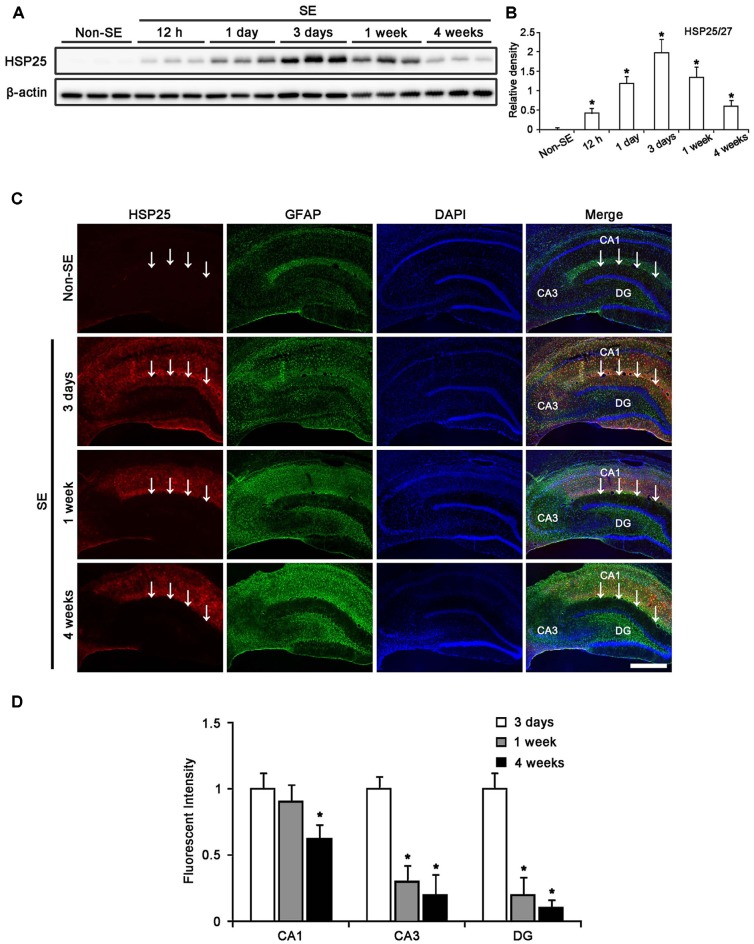
**Heat shock protein 25 (HSP25) expression in the hippocampus following status epilepticus (SE). (A)** Western blot (WB) shows the gradual up-regulation of HSP25 expression from 12 h to 3 days after SE. HSP25 expression is declined in the hippocampus from 1 to 4 weeks after SE. **(B)** Quantification of HSP25 expression level based on WB data (mean ± SEM; *n* = 7, respectively). *p < 0.05 vs. non-SE animals. **(C)** Double immunefluorescent images for HSP25 and GFAP in the hippocampus following SE. In non-SE animals, HSP25 immunoreactivity is rarely observed in the hippocampus. Three days after SE, HSP25 expression is increased in all hippocampal regions. One week after SE, HSP25 expression is reduced in the dentate gyrus (DG) and CA3 region as compared to non-SE animals. Four weeks after SE, HSP25 is also decreased in the CA1 region. Bar = 300 μm. Arrows indicate the hippocampal fissure (the upper border of the molecular layer of the DG). **(D)** Quantification of HSP25 fluorescent intensity in the hippocampus following SE (mean ± SEM; *n* = 7, respectively). *p < 0.05 vs. 3 days after SE.

Figure 1A shows that HSP25 band was detected from 12 h post-SE animals (*p* < 0.05 vs. non-SE animal). HSP25 protein expression increased until 3 days after SE (*p* < 0.05 vs. non-SE animal), and gradually decreased 1 and 4 weeks after SE (*p* < 0.05 vs. 3 days after SE; Figures 1A,B). Immunohistochemical study revealed that HSP25 expression was observed in astrocytes within the all hippocampal region 3 days after SE. One week after SE, HSP25 expression was reduced in the CA3 field and the DG (*p* < 0.05 vs. 3 days after SE; Figures 1C,D). Thus, HSP25 expression was mainly observed in astrocytes within the stratum radiatum of the CA1 field. Four weeks after SE, HSP25 expression was also declined in the CA1 field (*p* < 0.05 vs. 3 days after SE; Figures 1C,D). These findings indicate that HSP25 expression showed the distinct spatio-temporal specific pattern in the hippocampus following SE.

### Astroglial Death and HSP25 Induction in the Hippocampus Following SE

**Figure 2:**
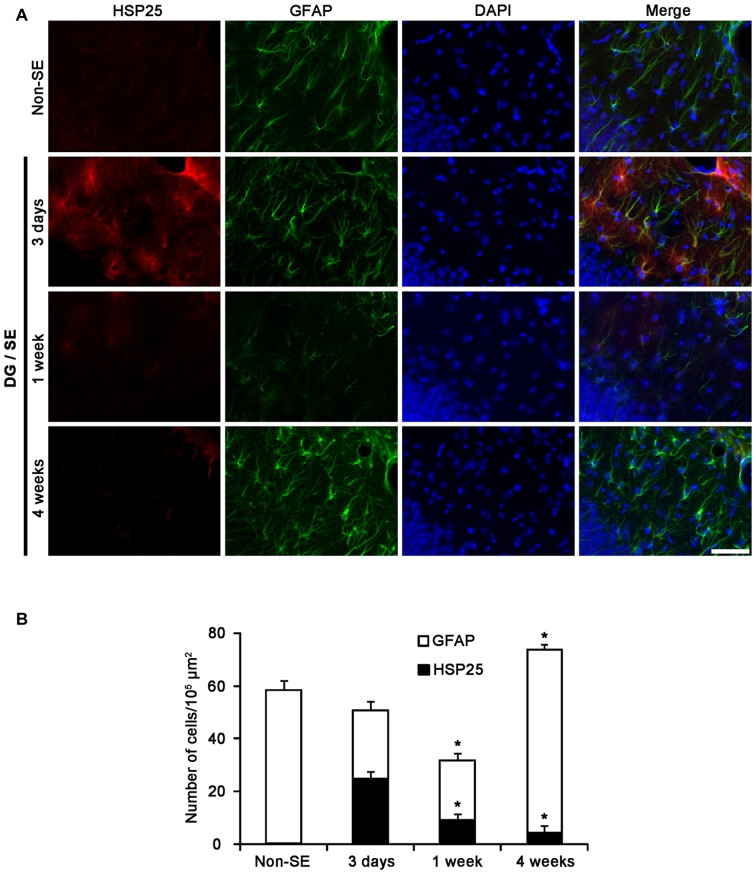
**HSP25 expression in astrocytes within the DG. (A)** Double immunofluorescence (IF) images for HSP25 and GFAP in the DG following SE. Three days after SE, HSP25 expression is up-regulated in the astrocytes in the molecular layer of the DG, as compared to non-SE animals. One week after SE, HSP25 expression is reduced in this region, accompanied by decline in GFAP expression. Four weeks after SE, HSP25 expression is rarely observed in reactive astrocytes. Bar = 50 μm. **(B)** The fraction of HSP25 positive astrocytes in total number of astrocytes within the molecular layer of the DG following SE (mean ± SD; *n* = 7, respectively). *p < 0.05 vs. 3 days after SE.

In the molecular layer of the DG, 50% of astrocytes showed HSP25 expression 3 days after SE (Figures 2A,B). One week after SE, total number of astrocytes was reduced as compared to 3 days after SE (*p* < 0.05, respectively; Figures 2A,B). The fraction of HSP25 positive astrocytes in total astrocytes was also declined to 9.3% in this region (*p* < 0.05, respectively; Figures 2A,B). Four weeks after SE, the fraction of HSP25 positive astrocytes in total astrocytes was decreased to 4.3%, since the total number of astrocytes was increased as compared to 3 days after SE (*p* < 0.05; Figures 2A,B).

**Figure 3:**
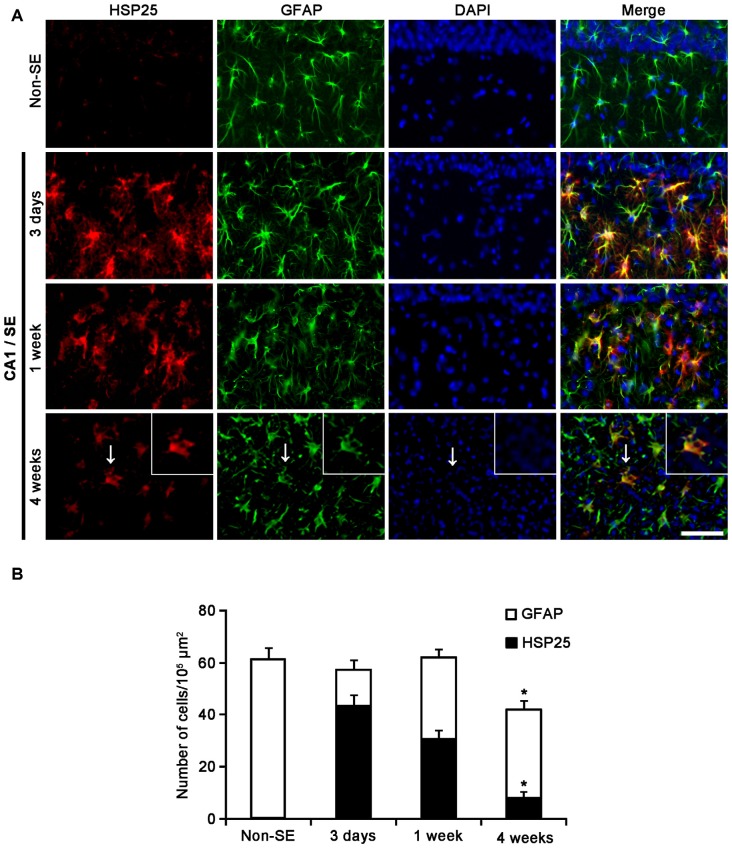
**HSP25 expression in astrocytes within the CA1 region. (A)** Double IF images for HSP25 and GFAP in the DG following SE. Three days and 1 week after SE, HSP25 expression is up-regulated in the astrocytes in the CA1 region, as compared to non-SE animals. Four weeks SE, HSP25 expression is mainly detected in clasmatodendritic astrocytes (arrow). Insertions in the lowest row are high magnification of arrows. Bar = 50 and 25 (insertion) μm. **(B)** The fraction of HSP25 positive astrocytes in total number of astrocytes in the CA1 region following SE (mean ± SD; *n* = 7, respectively). *p < 0.05 vs. 3 days after SE.

In the CA1 field, about 70% of astrocytes showed HSP25 expression 3 days after SE (Figures 3A,B). One week after SE, the fraction of HSP25 positive astrocytes in total astrocytes was 48% of astrocytes (Figures 3A,B). However, total number of astrocytes was unaltered. Four weeks after SE, total number of astrocytes was reduced in this region, as compared to 3 days after SE (*p* < 0.05; Figures 3A,B). The fraction of HSP25 positive astrocytes in total astrocytes was also decreased to 19% (*p* < 0.05 vs. 3 days after SE; Figures 3A,B). Interestingly, HSP25 was accumulated in clasmatodendritic astrocytes, which had round-shaped edematous cell body, short blunt processes, loss of distal processes, vacuoles, GFAP tangles (aggregation) and nuclear dissolution (absence of nucleus or watery pale nuclear staining), more than that in reactive astrocytes (Figure 3A). These findings indicate that the distinct HSP25 induction may be relevant to the different patterns of SE-induced astroglial loss in the hippocampus.

### Effect of HSP25 Knockdown on Seizure Susceptibility in Response to Pilocarpine

**Figure 4:**
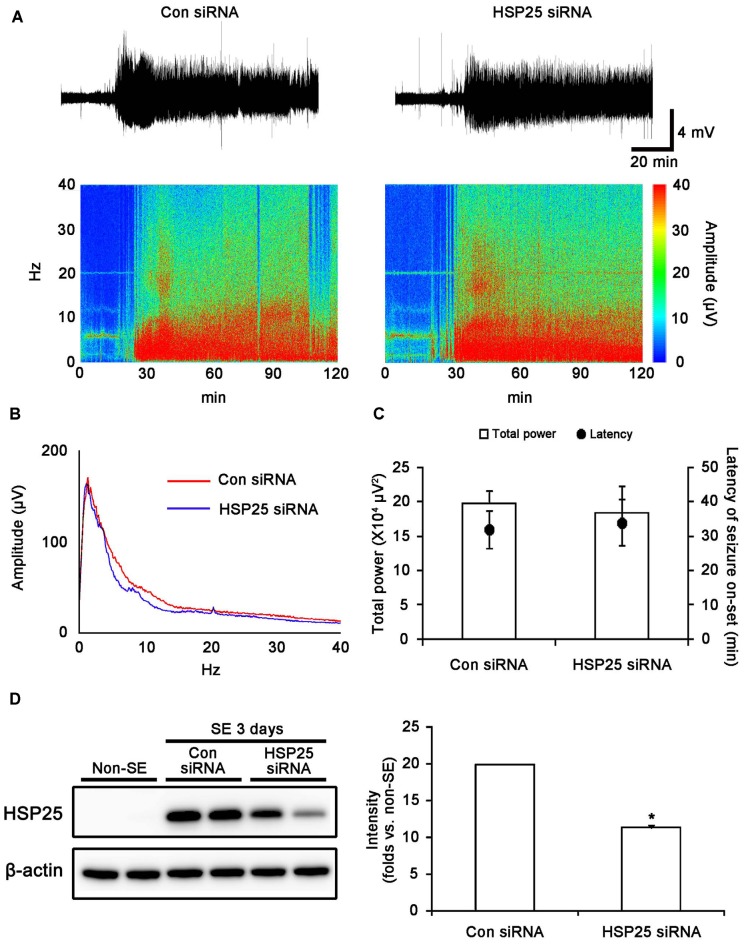
**Effect of HSP25 siRNA infusion on seizure susceptibility in response to LiCl-pilocarpine and SE-induced HSP25 expression. (A,B)** Representative EEG traces, frequency-power spectral temporal maps and spectrum view from control siRNA- and HSP25 siRNA-infused animals. **(C)** Total EEG power and latency of seizure on-set after pilocarpine injection. *p < 0.05 vs. control siRNA-infused animals (mean ± SD; *n* = 7, respectively). HSP25 knockdown did not affect the latency of seizure on-set and total power during SE. **(D)** Effect of HSP25 siRNA on SE-induced HSP25 expression. *p < 0.05 vs. control siRNA infusion (mean ± SEM; *n* = 7, respectively). HSP25 knockdown effectively reduces SE-induced HSP25 expression.

To investigate the role of HSP25 in SE-induced astroglial responses, we applied HSP25 knockdown before SE induction. Control- or HSP25 siRNA-infusion could not induce neurotoxicity (hind-limb paralysis, vocalization, food intake, seizure or neuroanatomical damage), indicating that both siRNA did not affect brain activity under normal condition. After SE induction, the first behavioral seizure occurred 32.1 and 34.3 min in control siRNA- and HSP25 siRNA-infused animals, respectively. In addition, both control siRNA- and HSP25 siRNA-infused animals showed episodes of high-amplitude and high-frequency discharges representing typical SE. An EEG analysis revealed no difference in the latency of seizure on-set and total EEG power during SE between control siRNA- and HSP25 siRNA-infused animals (Figures 4A–C). As shown in Figure 4D, HSP25 siRNA resulted in an approximate 43.2% reduction of HSP25 protein expression level following SE, as compared with control siRNA (*p* < 0.05). These findings indicated that HSP25 siRNA effectively inhibited SE-induced HSP25 induction without alterations in seizure susceptibility or severity in response to pilocarpine.

### Protective Role of HSP25 in Astroglial Apoptosis in the Molecular Layer of the Dentate Gyrus

**Figure 5:**
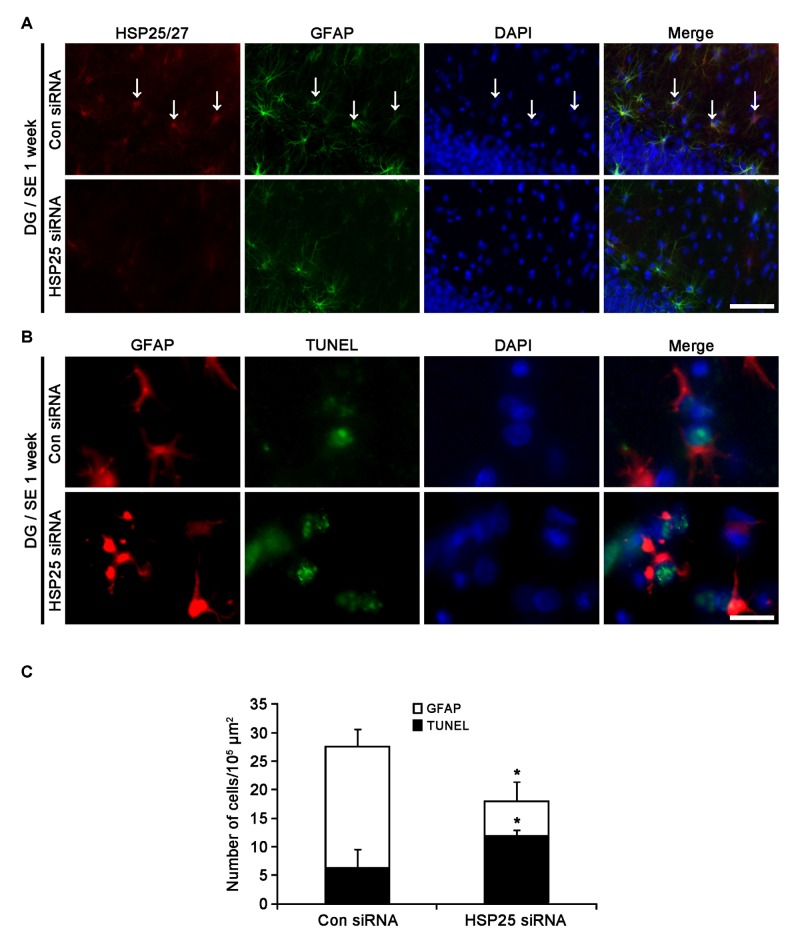
**Effect of HSP25 siRNA infusion on SE-induced astroglial death in the molecular layer of the DG 1 week after SE. (A)** Double immunofluorescent images for HSP25 and GFAP in the DG. HSP25 siRNA infusion effectively decreases HSP25 expression in astrocyte. Bar = 50 μm. Arrows indicate HSP25 positive astrocytes. **(B)** Double immunofluorescent images for transferase dUTP nick end labeling (TUNEL) and GFAP in the DG. As compared to control siRNA, HSP25 siRNA infusion increases the number of TUNEL positive astrocytes in this region. Bar = 12.5 μm. **(C)** The fraction of TUNEL positive astrocytes in total number of astrocytes (mean ± SD; *n* = 7, respectively). *p < 0.05 vs. control siRNA.

Next, we investigated the effect of HSP25 knockdown on SE-induced astroglial apoptosis in the DG using TUNEL staining. Consistent with our previous studies (Kang et al., 2006; Kim et al., 2010b), control siRNA-infused animals showed apoptotic astroglial degeneration in the DG 1 week after SE (Figures 5A–C). HSP25 siRNA exacerbated astroglial loss in this region, and increased the number of TUNEL positive astrocytes (*p* < 0.05 vs. control siRNA infusion; Figures 5A–C). Thus, it is likely that HSP25 may play an anti-apoptotic role in astrocytes within the DG.

### Protective Effect of HSP25 Knockdown on Clasmatodendritic Astroglial Loss in the CA1 Region

**Figure 6:**
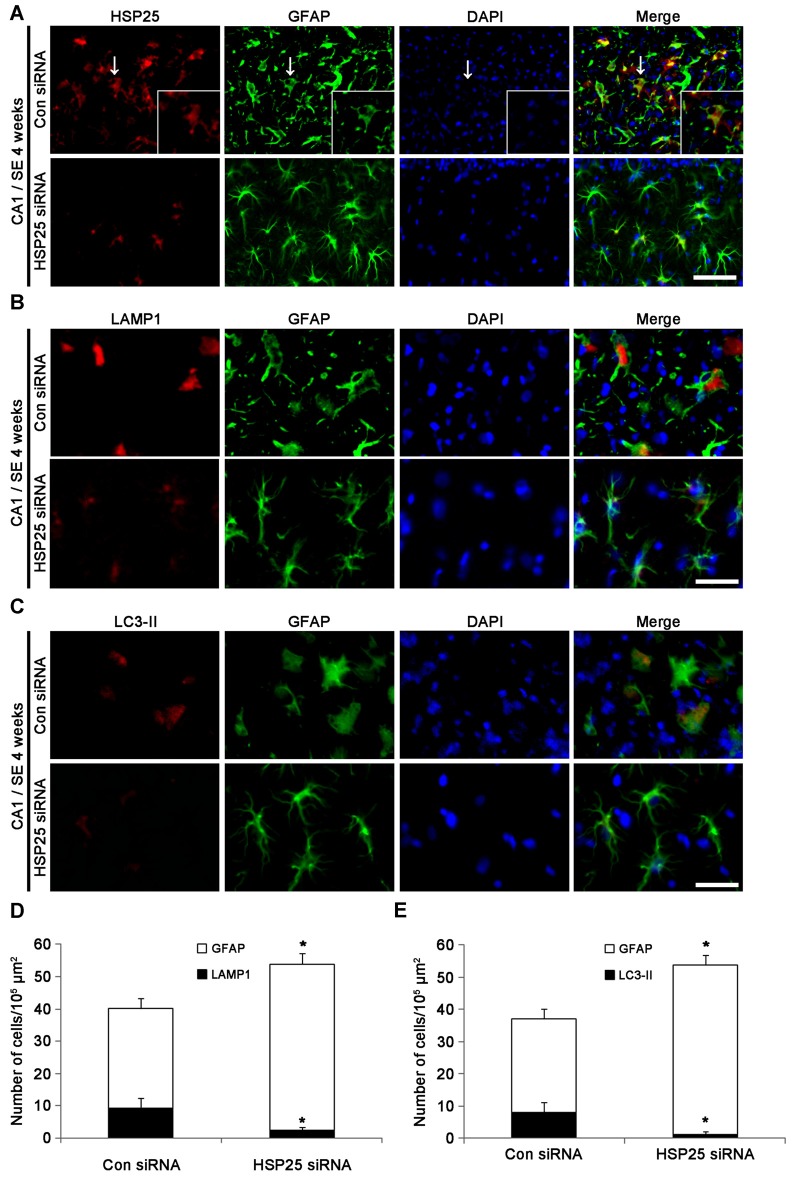
**Effect of HSP25 siRNA infusion on clasmatodendrosis in the CA1 region 4 weeks after SE. (A)** Double immunofluorescent images for HSP25 and GFAP in the CA1 region. As compared to control siRNA, HSP25 siRNA infusion decreases HSP25 expression in astrocytes. Insertions in upper row are high magnification of arrows. Bar = 50 and 25 (insertion) μm. **(B)** Double immunofluorescent images for lysosome-associated membrane protein 1 (LAMP1) and GFAP in the CA1 region. Bar = 20 μm. **(C)** Double immunofluorescent images for LAMP1 and GFAP in the CA1 region. Bar = 20 μm. As compared to control siRNA, HSP25 siRNA infusion effectively reduces the numbers of LAMP1 and LC3-II positive astrocytes in this region. Bar = 50 μm. **(D,E)** The fractions of LAMP1 **(D)** and LC3-II **(E)** positive astrocytes in total number of astrocytes (mean ± SD; *n* = 7, respectively). *p < 0.05 vs. control siRNA.

Since up-regulations of LAMP1 and LC3-II represent clasmatodendrosis (Ryu et al., 2011a,b), we applied double immunofluorescent studies for GFAP and LAMP1/LC3-II to evaluate the effect of HSP25 siRNA on autophagic astroglial death in the CA1 region. Four weeks after SE, control siRNA-infused animals showed clasmatodendrosis in the stratum radiatum of the CA1 region (Figure 6A). Consistent with our previous studies (Ryu et al., 2011a,b), clasmatodendritic astrocytes showed LAMP1-positive vacuolization and up-regulated LC3-II expression (Figures 6B,C). HSP25 siRNA effectively abrogated LAMP1-positive vacuolization and LC3-II over-expression. In addition, HSP25 siRNA prevented the decrease in the total number of astrocytes (*p* < 0.05 vs. control siRNA; Figures 6A–E). These findings indicate that sustained HSP25 expression may contribute to SE-induced autophagic astroglial death in the CA1 region.

### Reduced SE-Induced Astroglial ER Stress by HSP25 Knockdown

**Figure 7:**
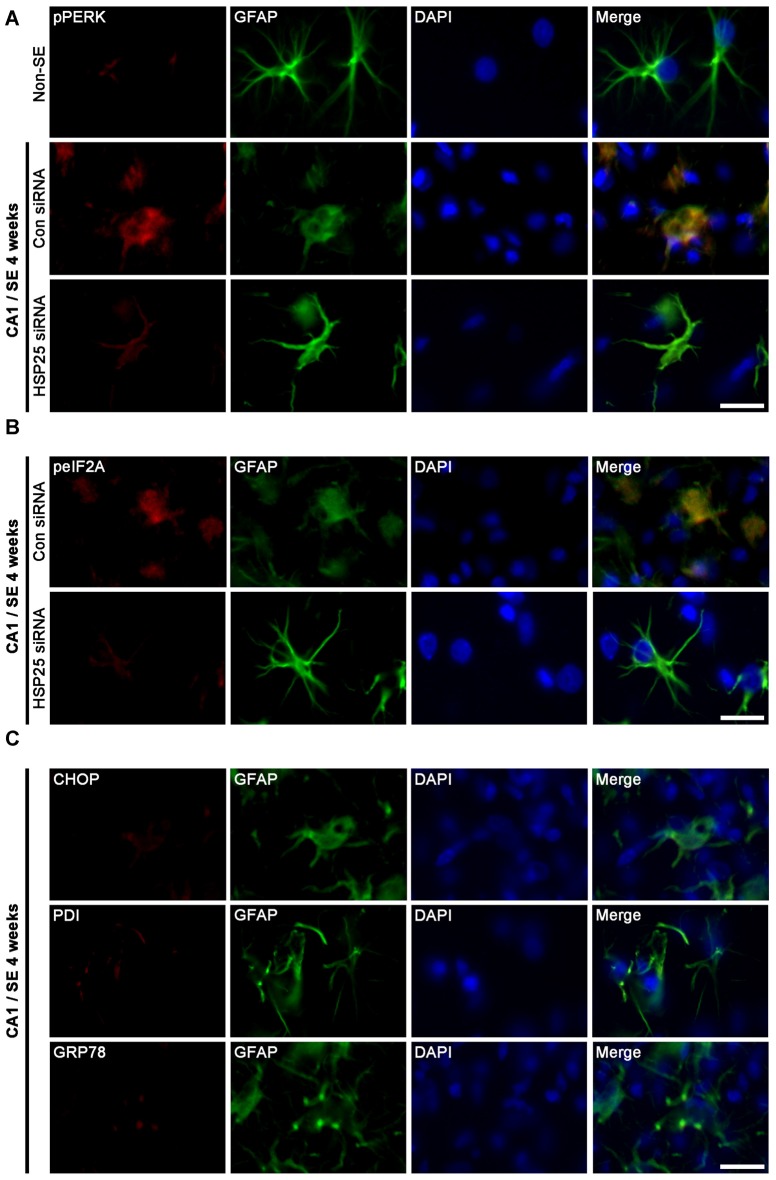
**Effect of HSP25 siRNA infusion on endoplasmic reticulum (ER) stress-related protein expressions in astrocytes within the CA1 region 4 weeks after SE. (A,B)** Double immunofluorescent images for phospho-PERK (pPERK), phospho-eIF2A (peIF2A) and GFAP in the CA1 region. Bar = 12.5 μm. pPERK and peIF2A levels are elevated in clasmatodendritic astrocytes, as compared to naïve astrocytes. HSP25 siRNA infusion effectively prevents phosphorylations of PERK and eukaryotic initiation factor 2α (eIF2A) in astrocytes and clasmatodendrosis. **(C)** Double immunofluorescent images for CCAAT/enhancer-binding protein homologous protein (CHOP), protein-disulfide isomerase (PDI), glucose-regulated protein 78 (GRP78) and GFAP in the CA1 region. Clasmatodendritic astrocytes do not show CHOP, PDI and GRP78 expressions Bar = 12.5 μm.

**Figure 8:**
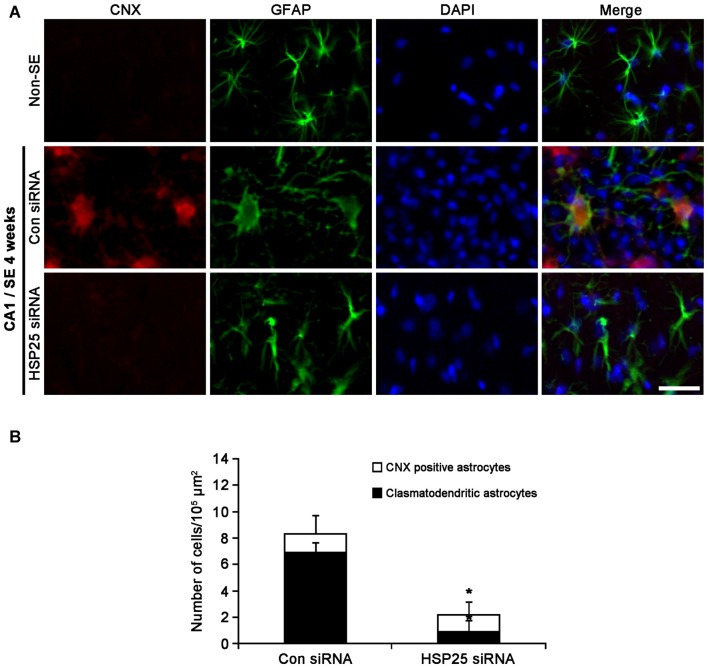
**Effect of HSP25 siRNA infusion on calnexin (CNX) expression in CA1 astrocytes 4 weeks after SE. (A)** Double immunofluorescent images for CNX and GFAP in CA1 astrocytes. In non-SE animals, CNX expression is rarely observed in CA1 astrocytes. Most of clasmatodendritic astrocytes show CNX expression. HSP25 siRNA infusion effectively prevents up-regulation of CNX expression in CA1 astrocytes and clasmatodendrosis. Bar = 20 μm. **(B)** The fraction of CNX positive astrocytes in total number of astrocytes (mean ± SD; *n* = 7, respectively). *p < 0.05 vs. control siRNA.

Protein kinase RNA (PKR)-like ER kinase (PERK) activation mediates the initial ER stress response by phosphorylating eukaryotic initiation factor 2α (eIF2A). In turn, the activation of the PERK/elF2A leads to ER stress-induced cell death via CCAAT/enhancer-binding protein homologous protein (CHOP)-dependent apoptosis (Moreno et al., 2012; Moreno and Tiffany-Castiglioni, 2015) or CHOP-independent autophagy (Bernales et al., 2006; Ito et al., 2009; Matsumoto et al., 2013). Recently, we have reported that clasmatodendritic astrocytes contained phospho-PERK (pPERK) and phospho-eIF2A (peIF2A) immunoreactivities without CHOP expression, and that up-regulated calnexin (CNX) expression is associated to the activation of autophagy leading to clasmatodendrosis (Ryu et al., 2011a,b; Ko et al., 2015). Therefore, it is likely that sustained HSP25 expression in CA1 astrocytes may be relevant to clasmatodendrosis via ER stress-mediated autophagy. To confirm this hypothesis, we investigated the effect of HSP25 knockdown on astroglial ER stress induced by SE. In non-SE animals, pPERK expression was rarely observed in CA1 astrocytes (Figure 7A). Following SE, clasmatodendritic astrocytes showed pPERK and peIF2A immunoreactivity, which was attenuated by HSP25 siRNA infusion (*p* < 0.05 vs. control siRNA; Figures 7A,B). In addition, most of clasmatodendritic astrocytes showed strong CNX expression without CHOP, protein-disulfide isomerase (PDI) or glucose-regulated protein 78 (GRP78) expressions (Figures 7C, 8A). HSP25 knockdown effectively decreased CNX expression in astrocytes as well as clasmatodendritic astroglial death (*p* < 0.05 vs. control siRNA infusion; Figures 8A,B). These findings indicate that accumulation of HSP25 protein may induce autophagic astroglial death via prolonged ER stress.

## Discussion

The major novel findings in the present study are that HSP25 regulates regional specific astroglial death in the hippocampus following SE. Briefly, HSP25 induction played an anti-apoptotic role in astrocytes within the DG, but initiated ER stress-mediated autophagic astroglial death in the CA1 region.

HSP25 is the main chaperone in astrocytes (FitzGerald et al., 2007), which plays a role in blockade of mitochondrial damage and apoptosis in naïve astrocytes (Bruey et al., 2000; Parcellier et al., 2003). In the present study, HSP25 induction was observed in the hippocampus 3 days after SE. These findings are consistent with previous studies demonstrating astroglial HSP25 induction in various SE models and temporal lobe epilepsy patients (Kato et al., 1999; Erdamar et al., 2000; Bidmon et al., 2004, 2008; Kirschstein et al., 2012). Unexpectedly, we found that HSP25 induction showed regional specific patterns in the distinct hippocampal region. Astroglial HSP25 expression was transiently induced in the DG, while it was sustained in the CA1 region. With respect to the distinctive properties of astrocytes independently of hemodynamics (Kim et al., 2014a), these findings indicate that the distinct capability of HSP25 induction may represent the differential properties of astrocytes in the hippocampus.

In the present study, 50% of astrocytes showed HSP25 expression in the DG 3 days after SE. One week after SE, apoptotic astroglial degeneration was accompanied by reduction in HSP25 expression in this region, which was exacerbated by HSP25 siRNA infusion. These findings indicate that HSP25 induction may contribute to astroglial invulnerability to apoptosis-inducing stresses. In contrast to the DG, the present study demonstrates that clasmatodendritic astrocytes showed sustained HSP25 expression 4 weeks after SE. Clasmatodendrosis is the result from energy failure and acidosis coupled to mitochondrial inhibition in Alzheimer disease and brain ischemia (Friede and van Houten, 1961; Kraig and Chesler, 1990; Hulse et al., 2001). In epilepsy, we have speculated that clasmatodendrosis might be relevant to extracellular acidosis during seizure activity (Kim et al., 2009, 2011). Indeed, conventional anti-epileptic drugs inhibit clasmatodendrosis in the CA1 region (Kim D. S. et al., 2008). Thus, it is likely that clasmatodendrosis may be a consequence of prolonged recurrent seizures causing sustained HSP25 induction. Since astroglial loss increases neuronal excitability in the hippocampus (Kang et al., 2006; Kim D. S. et al., 2008), clasmatodendrosis may increase excitatory output from the CA1 region and subsequently drive synchronous epileptiform discharges to the other brain regions.

Autophagy, originally described as a stress response to nutrient deprivation, is an important catabolic route for bulk degradation of aberrant organelles and protein aggregates by the formation of double-membrane vesicles known as autophagosomes, which ultimately fuse with lysosomes (autolysosomes) for degradation of contents by lysosomal proteases. However, excessive or unquenched autophagic process leads to non-apoptotic programmed cell death (type II programmed cell death) independent of caspase activity (Bursch et al., 1996; Tsujimoto and Shimizu, 2005; Gozuacik and Kimchi, 2007). In the present study, both LC3-II and LAMP1 intensities in HSP25-positive clasmatodendritic astrocytes were higher than those in reactive astrocytes, which were effectively abrogated by HSP25 siRNA infusion. Since up-regulations of LAMP1 and LC3-II expression represent the formations of autophagosomes and autolysosomes (Ryu et al., 2011a,b), these findings indicate that sustained HSP25 expression may lead to clasmatodendrosis in the CA1 region. Therefore, our findings indicate that HSP25 may be one of the pro-autophagic factors in CA1 astrocytes.

How does sustained HSP25 expression induce autophagic astroglial death? Recently, we have reported that ER stress is closely relevant to clasmatodendrosis. During ER stress, aggregated proteins segregate unfolded molecules and inhibit their interaction with other cellular components (Sherman and Goldberg, 2001). In addition, accumulation of aggregated proteins induces HSP25 to prevent further aggregation (Lee and Goldberg, 1998; Haslbeck, 2002; Goldbaum and Richter-Landsberg, 2004; Richter-Landsberg, 2007). Therefore, HSP25 protects astrocytes against harmful stress by non-native proteins (Goldbaum et al., 2009). However, impaired clearance of HSP25 reduces cell viability in astrocytes (Jänen et al., 2010). In the present study, clasmatodendritic astrocytes showed GFAP tangles and fibrous GFAP-positive fragments in the cytoplasm (GFAP aggregation) with over-expressions of CXN as well as HSP25. CNX is one of the ER chaperones, which retains unfolded or unassembled N-linked glycoproteins in the ER with ATP and Ca^2+^ (Ou et al., 1995; Williams, 2006). Thus, up-regulation of CXN is an indicative of ER stress that activates autophagy modulated by PERK/eIF2A activation independent of CHOP (Bernales et al., 2006; Granell et al., 2008; Ito et al., 2009; Korkhov, 2009; Matsumoto et al., 2013; Ko et al., 2015; Men et al., 2015). Interestingly, the present data demonstrate that HSP25 siRNA-infusion effectively abrogated increases in pPERK, peIF2A and CNX expression induced by SE. Thus, our findings suggest that sustained HSP25 expression/accumulation may trigger ER stress in CA1 astrocytes. Alternatively, HSP25-mediated nuclear factor-kappa B (NF-κB) activation may also involve clasmatodendrosis, since HSP25 over-expression increases NF-κB nuclear relocalization, DNA binding and transcriptional activity (Parcellier et al., 2003). Indeed, clasmatodendritic astrocytes show increase in NF-κB activity (Ryu et al., 2011b), which leads to ER stress and autophagy (Nivon et al., 2009; Prell et al., 2014). Therefore, prolonged HSP25 induction may switch autophagy concomitantly with ER stress via activation of NF-κB signaling pathway. Taken together, the present data suggest that the extensive clasmatodendrosis may be autophagic astroglial degeneration *per se* induced by sustained HSP25-mediated prolonged ER stress.

In conclusion, we provide novel evidence that HSP25 induction involves regional specific astroglial death induced by SE. In the DG, the early HSP25 induction may play an anti-apoptotic role in astrocytes. In the CA1 region, sustained HSP25 expression may result in ER stress, which would induce clasmatodendrosis. To the best of our knowledge, these findings propose for the first time the double-edge profiles of HSP25 in astroglial death following SE. Therefore, we suggest that the HSP25 may be one of the modulators for autophagic astroglial death as well as apoptosis.

## Author Contributions

J-EK and T-CK designed and supervised the project. T-CK performed the experiments described in the manuscript with J-EK, H-WH and S-JM. J-EK and T-CK analyzed the data and wrote the manuscript.

## Conflict of Interest Statement

The authors declare that the research was conducted in the absence of any commercial or financial relationships that could be construed as a potential conflict of interest. The reviewer DT and handling Editor declared their shared affiliation, and the handling Editor states that the process nevertheless met the standards of a fair and objective review.
